# Shrinking Giants: On the Feasibility of Downsizing Hepatocellular Carcinoma with Immunotherapy Prior to Liver Transplantation

**DOI:** 10.3390/jcm15103923

**Published:** 2026-05-19

**Authors:** Juraj Prejac, Domina Kekez, Hana Lučev, Borna Ćutić, Viktor Domislović, Vibor Šeša, Gordan Adžić, Marin Golčić

**Affiliations:** 1 Department of Oncology, University Hospital Centre Zagreb, Kišpatićeva 12, 10000 Zagreb, Croatia; dkekez@kbc-zagreb.hr (D.K.); hlucev@kbc-zagreb.hr (H.L.);; 2 University of Zagreb, School of Dental Medicine, Gundulićeva 5, 10000 Zagreb, Croatia; 3 Department of Gastroenterology and Hepatology, University Hospital Centre Zagreb, Kišpatićeva 12, 10000 Zagreb, Croatia; bcutic1@kbc-zagreb.hr (B.Ć.); vdomislo@kbc-zagreb.hr (V.D.);; 4 School of Medicine, University of Rijeka, 51000 Rijeka, Croatia; marin.golcic@uniri.hr; 5 Clinic for Tumors, Clinical Hospital Center Rijeka, 51000 Rijeka, Croatia

**Keywords:** hepatocellular carcinoma, immunotherapy, liver transplantation

## Abstract

**Background/Objectives**: Hepatocellular carcinoma (HCC) is a major cause of cancer-related morbidity and mortality, with incidence expected to increase. Liver transplantation is the most definitive curative option for early HCC, but many patients present beyond accepted transplant criteria, including the Milan criteria. Downstaging aims to reduce tumor burden and enable transplantation without compromising long-term outcomes. **Methods**: We reviewed the literature on liver transplantation, immune checkpoint inhibitors, immunotherapy–locoregional therapy combinations, and immune-related adverse events in HCC. **Results**: Immunotherapy-based strategies are emerging as downstaging approaches in selected patients. In advanced HCC, immune checkpoint inhibitor combinations have improved objective response rates compared with tyrosine kinase inhibitors, reaching approximately 20–36% in pivotal phase III trials. In the downstaging setting, early data suggest that immune checkpoint inhibitors, particularly with locoregional therapies, can achieve sufficient tumor regression to permit transplantation in patients initially beyond criteria. The ImmunoXXL trial reported successful downstaging and transplantation in all 16 patients treated with atezolizumab–bevacizumab, with 62.5% complete pathological responses, 2-year recurrence-free survival of 90%, and overall survival of 94%. The VITALITY study achieved successful downstaging in 75.6% of patients beyond Milan criteria, with 36.7% undergoing transplantation and 3-year post-transplant survival of 85%. However, pre-transplant immune checkpoint inhibitor exposure carries a clinically relevant risk of acute allograft rejection, reported in approximately 16–28% of transplanted patients. **Conclusions**: Immunotherapy-based downstaging before liver transplantation is promising but remains non-standard. Its use should be restricted to carefully selected patients within multidisciplinary protocols, as evidence remains limited by small cohorts, heterogeneous regimens, uncertain washout intervals, and rejection risk.

## 1. Introduction

### 1.1. Methods

This is a structured narrative review focused on the feasibility of expanding HCC criteria beyond transplantation thresholds to meet liver transplantation criteria. A comprehensive review of the literature was conducted using the PubMed/MEDLINE, Scopus, and Web of Science databases, focusing on keywords including “hepatocellular carcinoma” OR “HCC” AND “liver transplantation” OR “immune checkpoint inhibitors” OR “immunotherapy” OR “locoregional therapy combinations” OR “immune-related adverse events,” OR “graft rejection.” This review was not conducted as a formal systematic review, and, therefore, no predefined inclusion/exclusion criteria, PRISMA flow diagram, or formal risk-of-bias assessment were applied. This approach aimed to provide a clinically oriented synthesis of emerging evidence rather than a fully exhaustive systematic analysis. The article mainly emphasizes the potential for downsizing with immunotherapy, particularly multimodal treatments and liver transplantation in managing hepatocellular carcinoma. Articles were selected based on relevance to the topic and clinical applicability. Priority was given to clinical guidelines, prospective studies, randomized controlled trials, meta-analyses, and systematic reviews. Given the limited and evolving evidence base, retrospective studies, case series, and smaller studies were also included when deemed clinically informative.

### 1.2. Epidemiology and Risk Factors for HCC

Hepatocellular carcinoma (HCC) is a major contributor to the global cancer burden and remains one of the deadliest cancers worldwide. It is currently the sixth most diagnosed cancer and the third leading cause of cancer-related deaths [1,2]. Recent data show that approximately 865,000 new cases and 758,000 deaths occurred worldwide in 2022. HCC makes up about 75–85% of all primary liver cancers [2,3]. There is a strong male predominance in HCC. In most regions, incidence and death rates are two to three times higher in men than in women [1,2,4].

The global incidence of HCC closely mirrors mortality patterns. The mortality-to-incidence ratio is estimated at 0.86, indicating the aggressive nature of the disease, diagnosis at advanced stages, and limited treatment options for a large proportion of patients. As a result, the overall outlook remains poor, with a five-year relative survival rate of about 18% [3,5].

The development of HCC is strongly linked to several well-known environmental, infectious, and metabolic risk factors. A comprehensive global analysis published in 2022 estimated that 78.4% of HCC cases could be attributed to nine modifiable risk factors [1]. Infectious causes account for 65.9% of HCC cases. Behavioral and toxic exposures are the second most significant category (22.4%), followed by metabolic factors at approximately 19.7% [1]. Chronic infection with hepatitis B virus (HBV) or hepatitis C virus (HCV) remains the leading cause of HCC worldwide, with cirrhosis being the most important clinical risk factor, regardless of the underlying etiology. The annual risk of developing HCC in cirrhosis patients is estimated at 2–4% [6,7]. Other causes include aflatoxin exposure from contaminated food, heavy alcohol consumption, tobacco use, excess body weight, and type 2 diabetes [3,4].

### 1.3. The Epidemiologic Shift

The epidemiologic situation of HCC has shifted significantly over the past several decades. Between 1990 and 2022, the contribution of infection-related and behavioral risk factors declined globally, whereas the contribution of metabolic risk factors steadily increased [1]. A 2021 analysis estimated that hepatitis B accounted for 37.4% of liver cancer deaths worldwide, hepatitis C for 30.3%, alcohol consumption for 19.1%, MASLD-related disease for 8.5%, and other causes for 4.3% [8]. The global burden of HCC is expected to increase substantially. Population growth, population aging, and the increasing prevalence of obesity and diabetes are likely to be major drivers of this trend [9,10]. Recently published data indicate that the number of new cases could rise from approximately 905,000 to nearly 1,392,000 by 2040 [11,12]. Similarly, the number of deaths is predicted to increase by 56.4% between 2020 and 2040, potentially reaching 1.3 million annually [12].

### 1.4. Liver Transplantation and Patient Selection

When validated criteria for careful patient selection with HCC are used, liver transplantation (LT) remains the most definitive curative option available. Unlike other therapeutic options, LT provides dual therapeutic benefit by removing both the tumor itself and the cirrhotic liver in which it emerged. This is particularly relevant for patients with early-stage HCC who are unsuitable for resection because of impaired liver function, portal hypertension, or multifocal disease. In such patients, LT can achieve long-term outcomes, including median survival approaching 10 years and substantially lower recurrence rates than after resection or ablation. Five-year recurrence rates after LT are approximately 10%, compared with 50–60% after resection or ablation [6].

These outcomes depend primarily on rigorous patient selection. The Milan criteria, introduced by Mazzaferro and colleagues in 1996, remain the most widely used benchmark for determining transplant eligibility [6,7,13]. These criteria include patients with a single tumor measuring 1–5 cm or two to three tumors each measuring 1–3 cm, while ensuring there is no macrovascular invasion or extrahepatic disease [6,7]. Among patients fulfilling the Milan criteria, five-year overall survival rates of 75–80% and ten-year overall survival rates of 50–61.5% are commonly reported [3,14], with disease-free survival generally around 45–50% [7,13,15,16,17]. Because outcomes among Milan-criteria patients have been consistently good, several groups have explored whether slightly broader criteria could safely expand transplant eligibility [6].

Several models have been proposed:

1. University of California, San Francisco criteria (UCSF criteria): one tumor ≤ 6.5 cm or 2–3 tumors ≤ 4.5 cm with total tumor diameter ≤ 8 cm (approximately 81% five-year survival) [6,18].
2. Up to seven criteria: the sum of the largest tumor diameter (cm) and the number of tumors ≤ 7 (about 71% five-year survival) [6,14].
3. Extended Toronto criteria: no strict tumor size or number limit provided vascular invasion and extrahepatic disease are absent, and biopsy shows well-to-moderate differentiation (around 68% five-year survival) [6].
4. Kyoto criteria: ≤10 tumors with maximum diameter ≤ 5 cm and serum DCP ≤ 400 mAU/mL (approximately 65% five-year survival) [6].

More recently, the Metroticket 2.0 model has gained attention for incorporating AFP levels alongside tumor burden to estimate post-transplant survival [19,20]. In practical terms, applying expanded criteria such as UCSF may lead to an increase in the number of transplant candidates by 5–20% without compromising outcomes [14,20]. Considering these expanded approaches, it is important to emphasize clear contraindications to transplantation. These include macrovascular invasion involving the portal or hepatic veins, extrahepatic metastases, lymph node involvement, and severe comorbidities or functional limitations incompatible with transplantation [7,21].

HCC recurrence develops in approximately 10–15% of transplant recipients and remains the most common cause of death after transplantation [6,14]. For patients with recurrence, the prognosis is typically poor, with a median survival close to one year [6]. The lung is the most common site of recurrence (about 40%), followed by the liver (approximately 33%) [6]. Risk prediction tools, such as the “Risk estimation of tumor recurrence after transplant score” (RETREAT score), used at the time of transplantation, can estimate five-year recurrence risk [6].

### 1.5. Organ Allocation and Waiting List

In the United States, liver allocation is based on the MELD-Na scoring system. Patients who meet Milan criteria, or who have been successfully downstaged to meet them, may receive exception points after a mandatory six-month waiting period [6]. Since May 2019, these patients have received an exception score of MMaT-3 (median MELD at transplant minus three points) within their distribution area to reduce the unfair advantage HCC patients had over non-HCC patients in securing donor livers [6]. In contrast to the United States system, which has a standardized MELD exception policy for HCC, Eurotransplant countries apply different approaches to HCC prioritization. Transplantation networks across Europe use varying LT criteria for HCC patients, leading to significant inconsistencies in access and allocation [22,23]. A 2017 study analyzing Eurotransplant waiting list outcomes revealed a significant imbalance between patients with and without MELD exceptions. Patients with standard exceptions for HCC had a 22.6% negative outcome rate (death or delisting as too sick), compared to 34.3% for patients without exceptions, suggesting that HCC patients with exception points were advantaged compared to non-HCC patients [24]. There is no consensus in European policy on the usage of expanded criteria, and decisions are often made at the national or central level. Recent literature suggests European systems should shift from fixed selection and allocation criteria to more flexible benefit-based models that incorporate graft characteristics, response to locoregional treatment, tumor biology (AFP, growth rate), liver function (Child–Pugh, BCLC staging), and transplant benefit calculations [24].

### 1.6. Criteria for Downstaging and Patient Selection

Downstaging is considered for selected patients with HCC whose tumor burden exceeds conventional transplant criteria but remains potentially reducible to acceptable transplant limits. Appropriate candidates generally have liver-confined disease, absence of extrahepatic spread, no macrovascular invasion, preserved or acceptable liver function, and no major comorbidities that would preclude transplantation. Patients with rapidly progressive disease, uncontrolled AFP increase, vascular invasion, extrahepatic metastases, or poor performance status are generally unsuitable for downstaging with curative transplant intent [25,26].

The definition of acceptable tumor burden before downstaging varies across transplant programs. One of the most commonly used frameworks is the United Network for Organ Sharing downstaging criteria (UNOS-DS), which include a single lesion ≤ 8 cm, two to three lesions each ≤ 5 cm with a total tumor diameter ≤ 8 cm, or four to five lesions each ≤ 3 cm with a total tumor diameter ≤ 8 cm, in the absence of macrovascular invasion or ex-trahepatic disease [27].

Prospective evidence from the XXL trial and a national multicenter cohort supports downstaging as a valid therapeutic strategy, showing that selected patients initially exceeding Milan or even UNOS-DS criteria can achieve favorable post-transplant outcomes after successful downstaging [28,29,30]. Successful downstaging is commonly defined as achieving the Milan criteria before listing, although some programs apply broader post-downstaging criteria, such as the Hangzhou criteria, or use prognostic models to individualize transplant eligibility based on tumor burden, AFP, histology, and predicted post-transplant outcomes [31,32,33].

Treatment selection is generally guided by the Barcelona Clinic Liver Cancer (BCLC) staging system and treatment algorithm, but in the downstaging setting, it also requires individualized assessment of tumor burden and distribution, liver function, performance status, transplant eligibility, and local expertise [34]. Historically, downstaging and bridging have relied on locoregional therapies such as transarterial embolization (TAE), transarterial chemoembolization (TACE), transarterial radioembolization (TARE), ablation, and stereotactic body radiotherapy (SBRT) [35]. These approaches can also serve as bridging therapies in patients already listed for transplantation, with the aim of maintaining tumor control and preventing progression during the waiting period [36].

Assessment of downstaging response should go beyond dimensional tumor shrinkage and should integrate radiological response, biological behavior, and disease stability over time. Radiological response is most commonly evaluated using modified Response Evaluation Criteria in Solid Tumors (mRECIST), which assesses viable tumor tissue based on arterial-phase enhancement on contrast-enhanced imaging [37]. AFP dynamics provide an additional marker of tumor biology, with favorable responses commonly defined as AFP reduction to <500 ng/mL or a decrease of more than 50% from baseline values [38,39]. Rising AFP despite radiological stability should raise concern for biologically aggressive disease and may argue against proceeding directly to LT. In practice, many centers apply an observation period of approximately three months after successful downstaging to confirm stability before listing or transplantation, although firm recommendations are lacking.

Systemic therapy is not currently established as a standard downstaging strategy before LT. However, in patients who are not suitable candidates for locoregional therapy or who do not achieve adequate response with locoregional approaches, systemic treatment may be considered within clinical trials or prospective protocols. This remains particularly relevant in the context of emerging multimodal approaches, where transplant feasibility must be balanced against response durability, treatment-related toxicity, timing of LT, and post-transplant safety concerns [40,41,42].

## 2. Immunotherapy Prior to Liver Transplantation

### 2.1. PD-1/PD-L1 i CTLA-4 Inhibitors: Mechanism of Action, TME, Immunomodulation

The introduction of ICIs has drastically changed the treatment paradigm of both solid and hematological malignancies. Immune checkpoints are regulatory pathways that prevent excessive activation of the immune system and maintain self-tolerance. Cytotoxic T-lymphocyte-associated protein 4 (CTLA-4) and programmed death-1 (PD-1) are inhibitory checkpoints that regulate T-cell activity at different stages of the immune response. HCC promotes tumor immune escape by coordinated activation of these regulatory pathways.

CTLA-4 primarily acts early in lymphoid tissues during the priming phase of the immune response by competing with the co-stimulatory receptor CD28 for binding to CD80/CD86 on antigen-presenting cells (APCs), but with much higher affinity. Its binding reduces stimulatory signaling, leading to decreased T-cell proliferation, cytokine production, and clonal expansion [43]. Additionally, CTLA-4 plays an important role in regulatory T-cell (Treg)-mediated immunosuppression, where it suppresses effector T-cell responses by downregulating CD80/86 expression on APCs, inhibiting cytokine secretion, and reducing cytotoxic activity [44].

PD-1 is expressed on the surface of activated T cells, B cells, and other immune cells. Binding to its ligands, programmed death-ligand 1 (PD-L1) and programmed death-ligand-1 (PD-L2), negatively regulates the immune response by reducing T-cell proliferation, impairing cytokine production and cytotoxic granule release, ultimately leading to T-cell exhaustion and apoptosis [45].

In HCC, which most often develops in the context of chronic inflammation and cirrhosis, these pathways establish an immunosuppressive tumor microenvironment (TME). Tumor cells and immune cells within the TME, such as tumor-associated macrophages and M2 macrophages, express PD-L1 and other inhibitory signals driven by IFN-γ signaling and hypoxia-induced HIF-1α activity. Meanwhile, T-cells upregulate PD-1 and CTLA-4 checkpoints, leading to T-cell exhaustion and immune evasion. Tregs and myeloid-derived suppressor cells (MDSCs) accumulate in the TME and secrete immunosuppressive cytokines such as IL-10 and TGF-β, thereby further promoting an immunosuppressive environment [46]. High intratumoral PD-L1 expression is associated with more aggressive disease, higher recurrence rates after resection, and poorer prognosis [47].

Blocking the CTLA-4 pathway with anti-CTLA-4 antibodies (e.g., ipilimumab, tremelimumab) promotes tumor-reactive T-cell priming, while blocking the PD-1 pathway with anti-PD-1 antibodies (e.g., nivolumab, pembrolizumab) or anti-PD-L1 antibodies (e.g., atezolizumab, durvalumab) restores exhausted T-cell effector function. In addition to T-cell activation, checkpoint blockade can induce clonal expansion of tumor-specific CD8^+^ cytotoxic T lymphocytes and enhance intratumoral immune infiltration. Combined blockade offers synergistic antitumor effects; however, it also results in a higher occurrence of immune-related toxicities [48].

Another important mechanism in HCC is VEGF blockade, which not only has anti-angiogenic effects but can also reverse tumor-induced immunosuppression. Abnormal tumor vasculature leads to endothelial dysfunction and hampers immune cell trafficking; inhibiting it may promote vascular normalization and facilitate T-cell infiltration into tumor tissue by reducing MDSC and T-reg accumulation, encouraging dendritic cell maturation, and boosting effector T-cell infiltration [49].

Previously used systemic therapies like sorafenib had a low overall response rate (ORR) of about 5%. ICIs show a higher ORR, approximately 15% with monotherapy and around 30% with combination therapy (both ICI/ICI and ICI/VEGF) [48]. Additionally, the depth of response (DoR), defined as the percentage of tumor shrinkage from baseline, appears greater with ICIs than with sorafenib [50].

Although ICIs represent a significant advancement in HCC treatment, not all patients respond to therapy. HCC can be broadly classified into immune-inflamed, immune-excluded, and immune-desert phenotypes. The inflamed phenotype is characterized by high T-cell infiltration and a more favorable response to ICIs. The immune-excluded phenotype is characterized by T-cells trapped in the peritumoral stroma, unable to penetrate the tumor tissue due to TGF-β-mediated fibrosis and vascular abnormalities. In contrast, the immune-desert phenotype shows a complete lack of T-cell infiltration [51]. Nearly one-third of HCC patients harbor a gain-of-function mutation in the Wnt/β-catenin pathway, which hampers dendritic cell recruitment and effective T-cell priming, leading to an immune-desert phenotype and resistance to ICIs [52]. Additionally, the cause of chronic liver disease influences the response to ICIs. Patients with non-viral HCC, especially those with non-alcoholic steatohepatitis-induced HCC, tend to have poorer responses compared to patients with viral causes [53]. This may stem from the buildup of dysfunctional, nonresponsive CD8^+^PD-1^+^ T cells in a metabolically stressed liver environment, which impairs effective tumor surveillance and reduces the effectiveness of PD-1/PD-L1 inhibition.

The ability of immunotherapy-based strategies to induce substantial and durable tumor regression has raised growing interest in their potential role as downstaging tools before LT.

### 2.2. Downstaging Studies: Case Series and Clinical Trials Utilizing Immunotherapy Prior to Liver Transplantation

The application of ICIs as a downstaging or bridging strategy prior to LT for HCC has emerged as a promising therapeutic paradigm. This approach aims to reduce tumor burden in patients initially exceeding transplant eligibility criteria, thereby expanding the pool of candidates who may benefit from curative LT.

#### 2.2.1. Prospective Clinical Trials

The ImmunoXXL trial is the first prospective phase II study specifically designed to evaluate LT after atezolizumab-bevacizumab downstaging in patients with intermediate and advanced HCC who are beyond extended transplant criteria [54]. This landmark study enrolled 16 patients with significant tumor burden (median size: 6.5 cm, IQR: 3–8 cm), elevated AFP levels (median AFP: 283 ng/mL, IQR: 6–1080), and portal vein thrombosis in 50% of cases, features traditionally considered contraindications for transplantation. With regard to the small sample size in the study, the results showed that all patients achieved successful downstaging to transplant eligibility after a median of 4.7 months (IQR: 2.4–7.6). Explant pathology demonstrated remarkable responses: 10 complete pathological responses (62.5%) and 6 partial responses (37.5%). The median washout period from the last atezolizumab-bevacizumab administration to transplant was 57.5 days (IQR: 29–87). At a median follow-up of 16 months, the 2-year recurrence-free survival (RFS) was 90%, and OS was 94%. Pre-transplant immune-related adverse events (irAEs) occurred in 3 patients (19%), while post-transplant acute rejection developed in 4 patients (25%), all of which were clinically manageable [54]. Although ImmunoXXL is an important proof-of-concept study showing that LT after atezolizumab–bevacizumab downstaging is feasible in carefully selected patients with initially intermediate or advanced HCC, the results are based on a very small and highly selected transplanted cohort. The main limitations are its single-center design, lack of a control group, short follow-up, and the fact that the analysis primarily reports outcomes of patients who ultimately reached transplantation rather than the true intention-to-treat benefit of the overall strategy. In addition, the observed rate of acute rejection after prior immunotherapy highlights the need for caution, standardized washout intervals, and prospective validation before broader implementation outside expert transplant multidisciplinary team protocols.

The VITALITY study is the first multiregional United States evaluation of pretransplant ICI use, including 117 consecutive patients with HCC who were assessed for LT between 2016 and 2023 [55]. This intention-to-treat (ITT) analysis provides critical real-world evidence on the feasibility and outcomes of this approach. Among the cohort, 86 patients (73.5%) exceeded Milan criteria at presentation. Of these, 65 patients (75.6%) were successfully downstaged, with a median time of 5.6 months. Overall, 43 patients (36.7%) proceeded to transplantation, including 18 (15.4%) who were initially within the Milan criteria and 23 (19.7%) who were successfully downstaged from beyond the Milan criteria. Notably, 94% of patients received concurrent ICIs and locoregional therapies (LRT), reflecting contemporary multimodal practice patterns. The 3-year ITT survival rate was 71.1%, with no significant difference between patients initially within the Milan criteria and those beyond the Milan criteria (73.5% vs. 69.7%, *p* = 0.329). Post-transplant 3-year survival reached 85%. Independent predictors of waitlist dropout included tumor burden beyond Milan criteria, AFP doubling from baseline, and radiographic response. Post-LT rejection occurred in 7 patients (16.3%), with 6 having received their last ICI dose less than 3 months prior to transplantation, resulting in one graft loss [55]. However, the study was limited by its retrospective design, substantial heterogeneity in treatment combinations, timing of ICIs, and downstaging protocols, which further complicates interpretation and limits the strength of the conclusions.

#### 2.2.2. Systematic Reviews and Meta-Analyses

A comprehensive systematic review and meta-analysis by Liu et al. [56] included eight studies comprising 229 patients with hepatocellular carcinoma who received ICIs prior to LT as bridging or downstaging therapy. The analysis provided pooled estimates of key post-transplant outcomes. The pooled post-transplant allograft rejection rate was 19% (95% CI: 12–30%). When stratified by ICI class, rejection occurred in 24% of patients receiving PD-L1 inhibitors, 18% of those receiving PD-1 inhibitors, and 20% of patients treated with bispecific or combination immunotherapy regimens. Among patients who developed rejection, the complete recovery rate was 78% (95% CI: 59–97%), while graft loss occurred in 4% of cases (95% CI: 1–7%). Regarding oncologic outcomes, the HCC recurrence rate after transplantation was 24% (95% CI: 12–36%), with a median recurrence-free survival of 17.63 months (95% CI: 11.57–23.69). The included studies varied widely in design, patient selection, ICI regimens, and definitions of rejection and recurrence, introducing significant heterogeneity that affects the robustness of pooled estimates. The overall mortality rate was 8% (95% CI: 4–12%), while rejection-related mortality remained relatively low at 2% (95% CI: 0–5%). These findings suggest that although rejection risk is elevated relative to historical controls, most rejection episodes are manageable with appropriate immunosuppressive adjustments [56].

An international collaborative study that analyzed 386 patients with hepatocellular carcinoma who received ICI therapy in the peri-transplant setting integrated data from a systematic literature review and institutional registries. The study evaluated the impact of ICI exposure before and after LT on graft rejection and survival outcomes.

Overall, graft rejection rates did not significantly differ between patients receiving ICIs before transplantation (17.5%) and those treated after transplantation (22.1%) (*p* = 0.351). However, post-transplant ICI use was associated with substantially worse graft-related outcomes, including higher rates of graft loss or dysfunction (47.1% vs. 25.9%, *p* < 0.05) and rejection-related mortality (47.1% vs. 18.5%, *p* < 0.05) compared with pre-transplant ICI exposure. Importantly, in patients treated with ICIs prior to transplantation, longer washout intervals were associated with a significantly lower risk of graft rejection. Specifically, a washout period longer than 30 days reduced rejection risk (OR = 0.36, 95% CI: 0.18–0.72, *p* = 0.004), while intervals exceeding 1.5 drug half-lives were associated with an even greater reduction in risk (OR = 0.24, 95% CI: 0.12–0.50, *p* < 0.001). Furthermore, graft rejection occurring after transplantation in patients previously treated with ICIs was strongly associated with poorer OS (HR = 5.17, 95% CI: 2.21–12.24, *p* < 0.001), highlighting the clinical importance of careful patient selection and timing of ICI therapy in the peri-transplant setting [57].

High heterogeneity in some pooled analyses suggests caution when interpreting pooled effect sizes; study-level differences in design, patient selection, and assay methods likely contribute to this variability.

#### 2.2.3. Single-Center Case Series

One cohort study evaluated 7 transplant recipients who underwent neoadjuvant PD-1 blockade combined with lenvatinib, followed by LT [58]. The ORR was 71%, and the disease control rate (DCR) was 85% per mRECIST criteria. Biopsy-proven acute rejection (BPAR) incidence was 30%, with 2 deaths attributed to BPAR. This early series highlighted both the promising efficacy and the significant risk of rejection associated with this approach [58].

One other multicenter retrospective cohort study from 11 Chinese centers included 83 recipients who received pre-LT ICI therapy [59]. During a median follow-up of 8.1 months, 23 recipients (27.7%) developed allograft rejection, with 7 (30.4%) confirmed by liver biopsy. Multivariate analysis identified a time interval between the last ICI administration and LT (TLAT) of ≥30 days as an independent protective factor against rejection (OR = 0.096, 95% CI: 0.026–0.357, *p* < 0.001). Allograft rejection was an independent risk factor for OS (HR = 9.96, 95% CI: 1.006–98.610, *p* = 0.043) [59] (Table 1).

## 3. Systemic Treatment for Advanced HCC

### 3.1. Immunotherapy for Advanced HCC

HCC is a chemotherapy-resistant tumor, for which not many therapeutic options existed until 2008. and the SHARP trial, which positioned sorafenib, a tyrosine kinase inhibitor (TKI), as a first-line treatment [60]. Since then, until 2020. There have not been many breakthroughs regarding systemic therapy, besides lenvatinib, which has proved to be non-inferior to sorafenib [61].

Since 2020, three pivotal phase 3 trials have positioned immunotherapy-based regimens at the forefront of HCC treatment, comparing either an ICI-antiVEGF combination or an ICI-ICI combination to TKI. IMBrave150, HIMALAYA, and CheckMate 9DW used atezolizumab–bevacizumab, tremelimumab–durvalumab, durvalumab, and ipilimumab–nivolumab combinations, respectively, and all showed improved OS (overall survival), ORR (overall response rate), and CR (complete response) [62,63,64]. In these trials, only a minority of enrolled patients had intermediate-stage disease (BCLC B), whereas the majority presented with advanced or unresectable HCC. Data presented in Table 2.

Since the introduction of these combinations, new data have emerged regarding their potential not only to achieve longer disease control and, consequently, increased survival, but also to downstage HCC. According to the registration trials, one of the biggest differences compared with TKIs is the response rate, which ranges from 20.1% to 36% for ICI-based combinations, versus 5.1% to 13% for TKIs [62,63,64]. Notably, the ORR for CheckMate 9DW, which primarily used lenvatinib as a comparator, is higher in both arms than in other trials.

Until now, downstaging was reserved for patients who were amenable to locoregional treatment, used either in that setting or as “bridging” to transplantation. In general, patients with a greater depth of response tend to have longer OS, with those who achieve a complete response (CR) experiencing the longest OS [65,66]. According to long-term data, a subset of patients who achieve a partial response may be eligible for curative resection or locoregional therapies, primarily radiofrequency ablation (RFA) and TACE, thereby aligning their OS with that of complete responders. This is especially applicable to patients with intermediate-stage HCC. Although partial tumor response occurs in 20–30% of patients, only a subset of these patients undergoes conversion therapy due to tumor localization or size, and they tend to have worse outcomes [65,67,68,69].

Liver transplantation is a standard curative method in HCC [70], but the combination of immunosuppression for the prevention of allograft rejection after immunotherapy has been deemed controversial, considering that the combination can result in both allograft rejection and HCC recurrence, with fatal consequences.

Recently, there have been reports of successful LT after either single-agent immunotherapy [42,71] or immunotherapy-based combinations [72,73,74,75]. Additionally, pooled data analysis confirmed the potential of this treatment sequence when patients meet certain criteria, with high success rates [76]. Recently, a prospective observational study reported LT after downsizing with the atezolizumab–bevacizumab combination, highlighting the promise of this treatment in patients with a meaningful response [54].

Certain factors have been identified as vital for both preventing allograft rejection and HCC recurrence: the duration of immunotherapy-based treatment, the “washout period” of immunotherapy before LT, and meeting LT criteria. It seems that the timing of immunotherapy withdrawal may be relevant not only for avoiding allograft rejection but also for reducing recurrences. Although there have been cases of allograft rejection, they are usually manageable with escalation of immunosuppression and do not appear to affect OS. [42,54,76,77] In some of the reports, circulating tumor DNA (ctDNA) was monitored [74], raising a question whether it can be used as a stratification tool for transplant eligibility, similarly to how it could be used as a predictor for HCC aggressiveness and recurrence [78]. Both ctDNA and cfDNA have been investigated for early HCC diagnosis and response to therapy. Currently, most data concern the response to locoregional treatment, but some studies have also investigated its use in the context of systemic treatment, in which high ctDNA fractions, lack of ctDNA clearance, and newly detected hotspot mutations have been associated with worse prognosis [79,80]. If confirmed in prospective trials, ctDNA might serve as a useful tool for identifying patients at low risk of systemic disease who might be amenable to curative treatment.

A real-world study was conducted on 125 HCC patients with 721 plasma samples divided into four cohorts for post-transplant surveillance, post-resection surveillance, on-treatment response, and inoperable disease [81]. This study evaluated whether a personalized, tumor-informed 16-plex PCR-NGS ctDNA assay (Signatera) can predict relapse and monitor treatment response in hepatocellular carcinoma patients treated with curative-intent resection or liver transplantation. In the post-transplant group, 97.2% of patients who were ctDNA-negative during the 2–12 week MRD window remained negative on subsequent surveillance. On-treatment ctDNA trends correlated with imaging-based response assessments, and ctDNA showed greater sensitivity than AFP with a longer median lead time to recurrence detection (7.9 months vs. 2.2 months for AFP).

Currently, most data on curative interventions after immunotherapy-based regimens come from the atezolizumab-bevacizumab combination, as it was the first combination approved. While it further highlights other potential complications, the most important one being impaired wound healing, a known side effect of VEGF inhibitors, it would be imperative to understand the implications of other approved regimens, given that some might have even higher downstaging potential.

### 3.2. Combinations of Immunotherapy and Locoregional Therapies

Given the immunomodulatory effects on the tumor microenvironment, combining local techniques with ICI has the potential to produce synergistic effects and promising results in HCC treatment. The effect is primarily manifested in the reactivation of immune cells by ICI following the immunosuppressive effects of local procedures [50,82,83,84,85,86,87].

Transarterial chemoembolization remains the most commonly used locoregional procedure for hepatocellular carcinoma, with an expected survival of 20 to 25 months, and is still considered the international standard for intermediate-stage disease [3]. In 2024, the consensus panel of the Society of Interventional Radiology confirmed TACE as the standard of care for intermediate-stage HCC [88]. A retrospective study of patients who achieved a complete radiologic response to DEB-TACE found that most experienced disease recurrence, with the liver as the most common site [89].

The way TACE induces ischemia and cell death leads to upregulation of proinflammatory pathways and VEGF induction [83,90,91]. Furthermore, the procedure is associated with increased PD-L1 expression [92], which may enhance ICI antitumor activity [93]. However, due to an immune cell-rich microenvironment and heterogeneity in PD-L1 expression, post hoc trial results are controversial, and PD-L1 is not considered a reliable biomarker on its own [84].

Similar to TACE, transarterial radioembolization is a locoregional therapy that involves the intra-arterial administration of radioisotopes, most commonly yttrium-90 (Y-90). Unlike TACE, which relies on inducing ischemia, this method’s antitumor effect is primarily due to radiation [88,94,95]. That makes this method more suitable for patients with HCC and portal vein thrombosis, primarily segmental and lobar [96], or active when combined with anti-VEGF therapy and/or ICI [83].

#### 3.2.1. TACE

While both the HIMALAYA and IMbrave150 trials [62,63] included patients ineligible for locoregional therapy or resection, the phase III EMERALD-1 clinical trial offers insight into the absolute benefit of adding immunotherapy to standard treatment in intermediate-stage HCC. The trial enrolled patients with embolization-eligible, unresectable HCC and Child–Pugh A-B7 liver function [83]. PFS, the primary endpoint, was 15.0 months for patients who received durvalumab + bevacizumab (D + B) added to TACE, significantly longer than 10 months for those who received durvalumab (D) and 8.2 months for patients treated with TACE alone [83]. The ORRs (a secondary endpoint) for D + B, D monotherapy, and placebo with TACE were 44%, 41.0%, and 30%, respectively. The combination of D + B also resulted in the longest response duration at 22.1 months, compared to 14.0 months in the durvalumab group and 16.4 months in the placebo-only group.

The benefits of PFS and ORR were confirmed by several other randomized trials. The Phase III trial LEAP-012 showed an absolute PFS benefit of 4.6 months (14.6 vs. 10 months) with the combination of the PD-1 inhibitor pembrolizumab, lenvatinib, and TACE, compared with TACE alone [50,85]. ORR was also significantly higher in the combination group than in the placebo + TACE group, at 46.8% versus 33.3% per RECIST 1.1. A Phase III open-label trial, TALENTACE, compared adding atezolizumab + bevacizumab to TACE versus TACE + placebo [85]. PFS showed a clinically meaningful improvement (10.32 months vs. 6.37 months), although OS data are still immature. ORR was 81.3% in the atezolizumab + bevacizumab group, significantly higher than 49.1% in the placebo + TACE group. Partial responses increased significantly across all three trials, but the percentage of radiological complete responses (CR) per RECIST 1.1 remained low. In EMERALD-1, LEAP-012, and TALENTACE, CR was achieved in 3%, 3.4%, and 3.5% of patients in the combination arms [50,83,86]. Although disease control rate (DCR) has not been directly demonstrated, the available results suggest significant potential for this strategy as a bridging therapy to LT in patients with hepatocellular carcinoma.

The results of several meta-analyses, including retrospective single- and multi-center studies, were consistent with these findings, confirming the benefit of adding ICI to TACE for both short- and long-term outcomes [97,98,99]. The pooled ORR was significantly higher in the ICI-combination group, 46.6% versus 26.4%, with CR achieved in 8.4% of patients compared to 4.0% with TACE [99]. Additionally, adding ICI to the combination of TACE and TKIs proved superior to TACE and TKIs alone [99]. Also, although based on retrospective data, a large study showed that combining TACE with ICI + anti-VEGF is better than ICI + anti-VEGF alone for OS, PFS, and ORR in patients with advanced HCC [100]. These findings were confirmed by a study by Jiang WY et al. [101], which reported significantly better survival and a higher ORR (63.3% vs. 39.4%) with TACE combined with ICI + anti-VEGF therapy.

OS data from the EMERALD-1, LEAP-012, and TALENTACE studies have not yet demonstrated a statistically significant advantage of combination therapy (TACE + ICI + anti-VEGF) over TACE monotherapy [50,83,86,102]. In the LEAP-012 study, the 24-month OS rate was 75% in the combination group versus 69% in the control group (HR 0.80; 95% CI 0.57–1.11), but this did not meet the predefined threshold for statistical significance [50,102]. In the EMERALD-1 and TALENTACE studies, OS data are not yet mature, and the final results did not show a statistically significant difference, possibly due to subsequent systemic therapies after disease progression [83,86]. Although all three studies report a significant improvement in PFS, the meta-analysis by Wang et al. [87] confirms that the benefit in OS has not yet been statistically proven.

In the EMERALD-1 study, the most common high-grade adverse events in the durvalumab plus bevacizumab group were hypertension (6%), anemia (5%), acute kidney injury (4%), and proteinuria (4%). In the durvalumab plus placebo group, the most common adverse events were anemia (4%) and post-embolization syndrome (3%), whereas in the placebo group, the most common adverse event was post-embolization syndrome (4%). Treatment-related mortality was 0% (durvalumab + bevacizumab), 1% (durvalumab + placebo), and 2% (placebo) [83]. In the LEAP-012 study, the most common high-grade adverse events in the lenvatinib plus pembrolizumab group were hypertension (24%) and thrombocytopenia (11%). Deaths due to adverse events occurred in 2% of patients in the experimental group and <1% in the placebo group. Immunologic adverse events, such as hypothyroidism and pneumonitis, were more common with combination therapy, and 9% of patients had high-grade immunologic adverse events [50]. A meta-analysis including TALENTACE shows that combination therapy significantly increases the risk of high-grade adverse events (RR 1.88) and serious adverse events (RR 1.65) compared with TACE monotherapy [87]. A detailed summary of the key parameters from the phase 3 studies is presented in Table 3.

#### 3.2.2. TARE

Several single-arm prospective studies have demonstrated the effect of combining TARE and ICI. In a phase II study of 40 patients who were not candidates for curative surgical treatment, nivolumab was continued after radioembolization; the primary endpoint, ORR, was 30.6%; median OS was 16.9 months, and median PFS was 5.6 months [103]. A significant toxicity was observed with treatment-related serious adverse events noted in 14% of patients, including Stevens-Johnson syndrome, hepatitis E infection, fever, liver abscesses, and ascites. Even higher ORRs were reported in two other studies with nivolumab in phase II and durvalumab in phase I/IIa, respectively—41.5% by RECIST 1.1 and 83.3% by mRECIST criteria [104,105]. In the first of these studies, 5 out of 42 patients achieved downstaging to hepatectomy [104].

Even though there is still no proven benefit in OS, adding ICI to TACE is expected to result in tumor shrinkage in a significant proportion of patients. For advanced disease, this enables a shift to lower BCLC-stage disease and the introduction of other therapeutic options, including OLT. That being said, careful patient selection is required due to risks of immune-mediated liver injury and potential for post-transplant rejection.

#### 3.2.3. Ablation and SBRT

According to EASL and AASLD guidelines [7,34,35], ablative techniques, including thermal ablation, cryoablation, and irreversible electroporation, are reserved for early-stage HCC. As with TACE, these techniques also modulate the tumor microenvironment. Ablation stimulates activation of the antitumor immune system, including natural killer (NK) and T cells, dendritic cell (DC) infiltration, and the secretion of pro-inflammatory cytokines, while also inducing immunosuppressive mechanisms such as myeloid-derived suppressor cells (MDSCs), regulatory T cells (Tregs), blood enhanced growth factors (BEGF), and PD-1/PD-L1 expression [82,87,106].

Although data from clinical studies are limited, the combination with an anti-CTLA-4 agent has shown positive effects [107]. Tremelimumab, added to local therapies (TACE, RFA, or chemoablation, depending on disease stage), resulted in a partial response in 5 of 19 evaluable patients (26.3%). Furthermore, there was a marked reduction in HCV viral load, and biopsies confirmed an increase in CD8^+^ T cells. Another trial demonstrated activation of tumor-specific T cells and reduced T-cell clonality in patients treated with tremelimumab before RFA or MWA [108]. Indeed, two other clinical trials testing PD-1 blockade combined with ablation demonstrated improved 1-year relapse-free survival and OS in patients with recurrent HCC [109], and increased ORR and improved survival in patients with advanced HCC after sorafenib failure [110].

Stereotactic body radiotherapy (SBRT) delivers high doses of radiation in a small number of fractions and has proven to be an effective and safe therapeutic option in the treatment of HCC. Clinical studies demonstrate local tumor control rates of 90–95% with durable responses [111]. The incidence of severe side effects is relatively low and mainly related to the Child–Pugh status and the radiation dose to the uninvolved liver. Given the efficacy and non-invasiveness of SBRT, it is increasingly used and was included as an option in the ESMO therapeutic algorithm for BCLC A-stage HCC when resection or thermal ablation is not feasible [112]. Furthermore, in the BCLC B stage, SBRT is listed as a bridging option to OLT, and in the BCLC D stage, as a treatment for symptom control. In line with that, the European Association for the Study of the Liver (EASL) recommends SBRT for the treatment of unresectable HCC with curative intent when thermal ablation is not possible [35].

A single-arm, prospective trial in 21 patients with unresectable HCC treated with camrelizumab (anti-PD-1 antibody) combined with SBRT achieved an ORR of 52.4% as the primary endpoint. Median PFS and OS were 5.8 and 14.2 months, respectively [113]. The median radiation dose in the study was 40 Gy (30–50 Gy delivered over five fractions), and the median number of camrelizumab cycles was 5. No severe adverse events were recorded. A phase II, prospective trial, START-FIT, aimed to downstage HCC by using sequential TACE and SBRT followed by avelumab, with the proportion of patients deemed amenable to curative treatment as the primary endpoint [114]. The trial included 33 patients, of whom 42% achieved CR, while in 12%, curative therapy followed (either resection or radiofrequency ablation). One similar phase II prospective trial, START-FIT, using STRIDE, employed sequential TACE and SBRT, followed by the STRIDE ICI combination (single-dose tremelimumab with regular-interval durvalumab) [115]. Thirty-three patients were enrolled, with a median tumor size of 11.1 cm. Using mRECIST, a high ORR of 72.7% was observed, with 14 patients (42.4%) achieving complete remission. Grade ≥ 3 immune-related AE occurred in four patients (15%) with avelumab and four patients (12.1%) receiving the STRIDE protocol. Both trials included patients who were not amenable to curative resection and had Child–Pugh A or B liver function. A prospective multicohort study by Chiang et al. [116] included 33 patients from the previously mentioned START-FIT study and 30 patients who received nivolumab (90%) or pembrolizumab (10%) in addition to TACE and SBRT. Of the 63 patients, 47 (74.6%) had a response, including tumor reduction. CR was achieved in 46.0%, PR in 28.6%, SD in 9.5%, and disease progression in 15.9%. Among patients who achieved CR, OS was significantly prolonged, with a 3-year OS rate of 75.5%.

## 4. Immune-Specific Safety Considerations and Risks

### 4.1. Immune-Related Adverse Events

Immune-related adverse events can impact any organ system but are most frequently observed in the skin, gastrointestinal tract, liver, and lungs [117]. While most side effects are mild, serious adverse reactions are rare but can be life-threatening [118]. The most common serious side effect is colitis, which occurs in about 20% of patients receiving PD-1 blockade therapy, with 2–5% experiencing severe colitis. In patients treated with the CTLA-4 inhibitor, colitis occurs in around 40% of cases and is severe in 10–15%. Combination therapy with PD-1/PDL-1 and CTLA4 inhibitors results in a higher incidence of gastrointestinal side effects compared to either drug alone [119,120].

#### 4.1.1. Endocrine Toxicity

Endocrine toxicity from immunotherapy is also a common side effect. Thyroiditis occurs in 8% of patients receiving PD-1 therapy, while hypophysitis affects 6%, most often seen with ipilimumab in CTLA-4 therapy. Although rare, adrenal gland insufficiency and immune-related pancreatic damage require early detection to prevent severe outcomes, including death [121]. The incidence of acute adrenal insufficiency ranges from 1.5% to 2%, depending on the treatment protocol [122]. Diabetes mellitus and diabetic ketoacidosis can occur at a rate of up to 1% with any immunotherapy, but the incidence is highest (1,5%) with the combination of nivolumab and ipilimumab [35].

#### 4.1.2. Hepatic Toxicity

Immune-related liver damage is a common side effect of immunotherapy, characterized by elevated AST and ALT levels. The extent of liver damage is classified based on the increase in these enzymes: hepatocellular (ALT > −5-fold above ULN or R > 5), mixed (R > 2 to <5), or cholestatic (ALP > −2-fold above ULN or R < 2). The classification depends on how much ALT or ALP exceeds the upper limit of normal (ULN) or the serum ALT/ALP ratio (R value = [ALT/ULN]/[ALP/ULN]) [35]. The risk of immune-related hepatotoxicity is influenced by factors such as the type of immunotherapy, drug dosage, and underlying liver disease. Incidence is higher with combined PD-1/PDL-1 and CTLA4 therapy compared to monotherapy, although liver toxicity in combined therapy is less severe than in other organs. Furthermore, patients with liver cirrhosis undergoing HCC treatment are more likely to experience increased AST and ALT levels than those receiving other combined immunotherapies for different primary tumors [123].

Among patients with HCC treated with immunotherapy, hepatic toxicity incidence varies based on the drug and dosage. In the CHECKMATE 040 trial, patients treated with nivolumab (anti-PD-1 antibody) had a 15% incidence of any-grade ALT increase and a 6% incidence of grade 3 ALT elevation [124]. Similarly, in the KEYNOTE-224 trial, pembrolizumab (another anti-PD1 antibody) was associated with 9% of patients experiencing any-grade ALT increase and 4% experiencing grade 3 elevation [125]. Duffy and colleagues reported that combining tremelimumab with ablation led to a 19% rate of any-grade ALT elevation, with 9% experiencing grade 3 elevation [107]. Combination therapy with ipilimumab and nivolumab showed ALT increases of 8–16% for any grade and 0–8% for grade 3, with a higher incidence at higher ipilimumab doses [126].

Patients treated with the STRIDE protocol, which combines durvalumab and tremelimumab, experienced any-grade ALT increases in 9,3% of cases, with Grade 3 increases in 2,6% [63]. In the Imbrave 150 study involving atezolizumab and bevacizumab, 12% of patients showed any-grade ALT increases, and 2% experienced Grade 3 increases [127].

#### 4.1.3. Myocarditis and Myositis

Cardiotoxicity (myocarditis) is a rare side effect of immunotherapy, but potentially fatal [118]. According to the study by Mahmood [128] and colleagues, which analyzed data from a multicenter registry, the incidence of myocarditis in patients treated with immunotherapy was 1.14%, with symptoms typically appearing around 34 days after treatment initiation. As with other side effects, the incidence was higher among patients receiving combined immunotherapy than among those receiving monotherapy. Notably, pembrolizumab as a standalone treatment had a higher rate of myocarditis than other monotherapies. Of those who developed myocarditis, 46% suffered permanent consequences, and more than half experienced a severity grade of 3 or above.

Similar to myocarditis, myositis is a rare complication of immunotherapy. In the study by Jayan et al. [129], myositis was recorded in only 0.4% of cases, again more frequently with combination therapy, but it can also occur with monotherapy (RR 3.1 for combination therapy). When using monotherapy, PD-1/PDL-1 has a higher incidence rate of myositis than CTLA4 therapy [130]. Myositis can present in combination with myocarditis and/or myasthenia gravis [131].

#### 4.1.4. Pneumonitis

Pneumonitis linked to immunotherapy is a rare but serious adverse effect. Like other side effects, combination therapy has the highest risk of pneumonitis, while monotherapy carries a lower risk. Among the treatments, atezolizumab has the lowest risk of pneumonitis, whereas ipilimumab alone has the highest [117].

#### 4.1.5. Dermatologic Toxicity

Dermatologic irAEs (dirAEs) are among the most common and often the earliest to occur in both adjuvant and metastatic settings [132]. They affect 30–50% of patients receiving immunotherapy and display a wide range of clinical features. These can include mild maculopapular rashes, psoriasis, and pruritus, as well as more severe conditions like autoimmune blistering diseases, pigmentary changes, and lichenoid eruptions [133,134]. The type of skin-related side effects often depends on the duration of therapy, with early irAEs such as pruritus and maculopapular rash appearing soon after treatment begins, and late irAEs like vitiligo, bullous pemphigoid, psoriasis, and lichen planus developing over time. Dermatologic irAEs usually remain mild to moderate but can become chronic and require extended treatment.

### 4.2. Graft Rejection and Optimal Washout Period

The administration of ICIs prior to LT poses a unique immunological challenge: the persistence of activated T cells that recognize allograft antigens, which can precipitate acute cellular rejection. Understanding the magnitude and determinants of this risk is essential for safe implementation of neoadjuvant immunotherapy strategies.

Pooled data from the meta-analysis by Liu et al. demonstrate a post-LT allograft rejection rate of 19% (95% CI: 12–30%) among patients receiving pre-LT ICIs [56]. This represents an elevation compared to historical rejection rates in HCC transplant recipients not exposed to ICIs, which typically range from 10 to 15. The clinical significance is tempered by the high complete recovery rate (78%) and low graft loss rate (4%) [56]. The VITALITY study reported rejection in 7 of 43 transplanted patients (16.3%), with 6 of these patients having received their last ICI dose less than 3 months prior to LT [55]. This temporal association underscores the importance of adequate washout periods. Similarly, the multicenter Chinese cohort by Guo et al. documented rejection in 27.7% of recipients, with rejection identified as an independent risk factor for mortality (HR = 9.96) [11].

#### 4.2.1. Rejection by ICI Class

Analysis of rejection rates by ICI class reveals modest differences: PD-L1 inhibitors: 24%; PD-1 inhibitors: 18%; bispecific/combination therapies: 20%; anti-CTLA-4 monotherapy: 25% [56,135]. The ASCO 2025 meta-analysis of solid organ transplant recipients found that anti-PD-L1 agents were associated with the lowest rejection rates (0% in liver transplant recipients), though sample sizes were limited [9]. Anti-CTLA-4 therapy demonstrated a protective effect against rejection (OR = 0.22), potentially due to its mechanism of action on regulatory T cells [135].

#### 4.2.2. Predictors of Allograft Rejection

A multicenter retrospective cohort study by Fang et al. (2026) identified immune-related adverse events (irAEs) as the strongest predictor of post-transplant rejection (OR = 9.17, *p* < 0.001) [136]. Patients experiencing irAEs during ICI therapy demonstrated significantly elevated peripheral CD8^+^ T cell counts and higher serum levels of IFN-α and TNF-α, suggesting a heightened state of immune activation that persists into the post-transplant period [135]. Additional independent risk factors identified include recipient age of <40 years (OR = 3.03), a 30-day washout period (OR = 3.07), and high PD-L1 expression on graft tissue (*p* < 0.001) [57,136].

#### 4.2.3. Optimal Washout Period Between Last ICI Dose and Transplantation

Determining the optimal interval between the last ICI administration and LT requires balancing tumor control with the risk of rejection. Current evidence supports several key recommendations. The American Association for the Study of Liver Diseases recommends discontinuing ICIs at least 3 months (90 days) prior to LT, while acknowledging that additional safety data are needed for use closer to transplantation [6]. This conservative recommendation reflects the limited prospective data available at the time of guideline development. Kuo et al. [77] analyzed 22 patients receiving atezolizumab, nivolumab, or pembrolizumab, all of which have a half-life of approximately 28 days. The study identified 42 days (1.5 half-lives) as the shortest safe washout period associated with significant rejection-free survival. Patients with severe rejection had a median washout period of only 22 days (IQR: 9–35 days) [77] (Table 4).

Based on the totality of the evidence, a minimum washout period of 6–8 weeks (42–60 days) appears to substantially reduce the risk of rejection while maintaining acceptable tumor control [57,77,135]. However, the more conservative 90-day interval recommended by AASLD provides an additional safety margin and remains appropriate for patients with stable disease [6]. The global cohort study by Ma et al. demonstrated that washout periods exceeding 1.5 half-life counts (approximately 42 days for most PD-1/PD-L1 inhibitors) were associated with a 76% reduction in rejection risk (OR = 0.24, 95% CI: 0.12–0.50) [57]. The ASCO 2025 meta-analysis further supported extended washout, showing that intervals > 60 days reduced the risk of rejection by 90% (OR = 0.10) [135].

Importantly, these washout thresholds should be contextualized by ICI class and therapeutic regimen. Although the pharmacokinetic half-lives of PD-1, PD-L1, and CTLA-4 inhibitors are broadly comparable (~14–28 days), the risk of rejection appears to differ by mechanism of action. The ASCO 2025 meta-analysis reported rejection rates of 40.6% for anti-PD-1, 25% for anti-CTLA-4, and 0% for anti-PD-L1 agents [9]. Kuo et al. identified 42 days (1.5 half-lives) as the minimum safe washout for PD-1/PD-L1 monotherapy, while Guo et al. reported that a washout ≥ 30 days significantly reduced rejection in patients receiving PD-1 inhibitors ± TKIs ± locoregional therapy (OR = 0.096) [59,77]. Ma et al. confirmed that washout exceeding 1.5 half-lives reduced rejection by 76% regardless of ICI class [57]. Schnickel et al. and Wassmer et al. endorsed a more conservative ≥ 90-day interval across all ICI classes [13,15].

## 5. Limitations of the Available Evidence

The available evidence on immunotherapy-based downstaging before LT remains limited and should be interpreted cautiously. Although several reports suggest that ICIs may enable LT in selected patients with HCC, the strength of the evidence differs substantially across studies. ImmunoXXL provides important prospective proof-of-concept data, but it included only 16 patients, was conducted in a highly selected cohort, lacked a control group, and had relatively short follow-up. In addition, the study mainly reflects outcomes among patients who ultimately reached transplantation, which may overestimate the real-world intention-to-treat benefit of this strategy [54]. The VITALITY study provides broader real-world data, including 117 patients assessed for LT, but its retrospective design, heterogeneous ICI regimens, frequent combination with locoregional therapies, and variable timing of treatment and transplantation limit causal interpretation [55].

Pooled analyses and registry studies are also affected by major heterogeneity. The meta-analysis by Liu et al. included only 229 patients across eight studies, with substantial variation in patient selection, ICI class, combination strategies, use of locoregional therapy, definitions of rejection and recurrence, and duration of follow-up [56]. Similarly, the international collaborative cohort of 386 patients combined literature-derived and institutional registry data, including both pre- and post-transplant ICI exposure, which increases sample size but introduces important heterogeneity in treatment context, timing, immunosuppression, and outcome reporting [57]. These factors limit the robustness of pooled estimates and make direct comparison between studies difficult.

The evidence from smaller case series should be viewed as hypothesis-generating rather than practice-changing. For example, the single-center cohort of seven patients treated with PD-1 blockade plus lenvatinib reported a high objective response rate, but also a 30% rate of biopsy-proven acute rejection and two rejection-related deaths [58]. The multicenter Chinese cohort of 83 recipients provided valuable information on rejection risk and timing from last ICI administration to LT, but follow-up was short, treatment regimens were heterogeneous, and rejection was biopsy-confirmed only in a subset of patients [59]. These studies highlight both the potential efficacy and the clinically relevant transplant-specific risks of this approach.

The optimal washout interval between the last ICI dose and LT remains uncertain. Available data suggest that longer washout periods reduce rejection risk, with proposed thresholds ranging from ≥30 days to ≥90 days and some analyses supporting intervals exceeding 1.5 drug half-lives [57,59,77,135]. However, these thresholds are derived mainly from retrospective cohorts, pooled analyses, and expert recommendations rather than prospective validation. Washout timing, therefore, remains individualized and should consider ICI class, regimen complexity, tumor control, liver function, prior immune-related adverse events, transplant urgency, and local transplant practice.

Finally, the present review is narrative rather than systematic, which may introduce selection bias in study inclusion and interpretation. Across the field, the lack of standardized eligibility criteria, response assessment, transplant timing, immunosuppression protocols, and long-term reporting of oncologic and graft-related outcomes remains a major barrier to clinical implementation. Prospective registries and controlled studies are needed to define which patients benefit most from ICI-based downstaging, how response should be assessed, what washout interval is safest, and how rejection risk should be minimized before this strategy can be incorporated into routine transplant algorithms.

## 6. Conclusions

Immunotherapy-based downstaging before liver transplantation is a promising but still investigational strategy for carefully selected patients with HCC beyond conventional transplant criteria. Early data suggest that ICIs, particularly as part of multimodal treatment, can induce meaningful tumor regression and enable LT in selected cases. However, potential benefit must be balanced against immune-related toxicity, adequate washout timing, and the risk of post-transplant rejection. Prospective trials and standardized registries are required before this approach can be incorporated into routine transplant algorithms.

## Figures and Tables

**Table 1:** Overview of major clinical trials and key parameters of systemic immunotherapy approaches for downstaging or bridging prior to liver transplantation in HCC.

Study	Design	N	Therapeutic Regimen	Downstaging Rate	Transplant Rate	Rejection Rate	Survival Outcomes
ImmunoXXL [54]	Prospective Phase II	16	Atezolizumab + bevacizumab (94% with prior/concurrent LRT)	100%	100%	25%	2-yr RFS 90%, OS 94%
VITALITY [55]	Multicenter Retrospective	117	Heterogeneous ICIs (94% with concurrent LRT); nivolumab, atezolizumab + bevacizumab, pembrolizumab, others	75.6%	36.7%	16.3%	3-yr ITT 71.1%, post-LT 85%
Meta-analysis [56]	Systematic Review	229	Heterogeneous ICIs (PD-1, PD-L1, bispecific/combination agents) ± LRT	—	—	19%	Median RFS 17.63 mo
Global Cohort [57]	International Registry	386	Heterogeneous ICIs (PD-1, PD-L1, CTLA-4 inhibitors) ± LRT; pre-LT and post-LT subgroups	—	—	17.5% (pre-LT)	—
Qiao 2021 [58]	Single-center Cohort	7	PD-1 inhibitor (toripalimab or camrelizumab) + lenvatinib	ORR 71%	100%	30%	—
Guo 2024 [59]	Multicenter Retrospective	83	PD-1 inhibitors (various) ± TKIs ± LRT; TLAT ≥ 30 days as protective factor	—	—	27.7%	—

Abbreviations: ICIs, immune checkpoint inhibitors; LRT, locoregional therapy; TKIs, tyrosine kinase inhibitors; TLAT, time from last anti-PD-1/PD-L1 therapy to liver transplantation; PD-1, programmed cell death protein 1; PD-L1, programmed death-ligand 1; CTLA-4, cytotoxic T-lymphocyte-associated protein 4; ORR, objective response rate; RFS, recurrence-free survival; OS, overall survival; ITT, intention-to-treat; LT, liver transplantation.

**Table 2:** Phase III trials demonstrating positive outcomes for immunotherapy based regimens.

Study	Design	N	Child Pugh Stage A, %	BCLC B, %	mOS, Months	mPFS, Months	ORR by RECIST1.1, %	TRAEs, Any Grade/Grade 3/4/Grade 5, %	AEs Leading to Discontinuation, %
IMBrave150 [62]	atezolizumab + bevacizumab vs. sorafenib	336 vs. 165	100 vs. 100	15 vs. 16	19.2 vs. 13.4	6.9 vs. 4.3	27.3 vs. 11.9	98.2 vs. 98.7/56.5 vs. 55.1/4.6 vs. 5.8	15.5 vs. 10.3
HIMALAYA [63]	tremelimumab + durvalumab vs. durvalumab vs. sorabenib	393 vs. 389 vs. 389	98.5 vs. 97.7 vs. 99.4	19.6 vs. 20.6 vs. 17.0	16.4 vs. 16.6 vs. 13.8	3.78 vs. 3.65 vs. 4.07	20.1 vs. 17.0 vs. 5.1	75.8 vs. 52.1 vs. 84.8/25.8 vs. 12.9 vs. 36.9/2.3 vs. 0 vs. 0.8	8.2 vs. 4.1 vs. 11.0
CheckMate 9DW [64]	ipilimimab + nivolumab vs. lenvatinib/sorafenib	335 vs. 333	97 vs. 96	27 vs. 26	23.7 vs. 20.6	9.1 vs. 9.2	36 vs. 13	83 vs. 91/41 vs. 42/0 vs. 0	18 vs. 10

Abbreviations: BCLC, Barcelona Clinic Liver Cancer; mOS, median Overall Survival; mPFS, median Progression-free Survival; ORR, Overall Response Rate; RECIST, Response Evaluation Criteria in Solid Tumors; TRAE, Treatment-Related Adverse Event; AE, Adverse Event.

**Table 3:** Phase III trials demonstrating positive outcomes for combined immunotherapy and TACE in intermediate-stage HCC.

Study	Design	N	Child Pugh Stage A, %	BCLC B, %	mOS, Months	mPFS, Months	ORR by RECIST1.1, %	TRAEs, Any Grade/Grade 3/4/Grade 5, %	AEs Leading to Discontinuation, %
EMERALD-1 [83]	TACE + durvalumab + bevacizumab vs. placebo + TACE	204 vs. 205	98 vs. 98	57 vs. 60	-	15.0 vs. 8.2 (HR= 0.77; *p* = 0.032)	44 vs. 30	81 vs. 45/27 vs. 6/0 vs. 2	28 vs. 8
LEAP-012 [85]	TACE + pembrolizumab + lenvatinib vs. placebo + TACE	237 vs. 243	100 vs. 100	57 vs. 60	75.0 vs. 69.0 (HR = 0.80; *p* = 0.087)	14.6 vs. 10.0 (HR = 0.66; *p* = 0.0002)	47 vs. 33	99 vs. 85/70 vs. 31/2 vs. <1	33 vs. 7
TALENTACE [86]	TACE + atezolizumab + bevacizumab vs. observation	171 vs. 171	100 vs. 100	59 vs. 61	34.5 vs. 35.4 (HR = 0.96)	11.3 vs. 7.0 (HR = 0.71; *p* = 0.009)	49 vs. 34	100 vs. 98/61 vs. 41/3 vs. 2	21 vs. 2

Abbreviations: TACE, transarterial chemoembolization; BCLC, Barcelona Clinic Liver Cancer; mOS, median Overall Survival; mPFS, median Progression-free Survival; ORR, Overall Response Rate; RECIST, Response Evaluation Criteria in Solid Tumors, TRAE; Treatment-Related Adverse Event; AE, Adverse Event.

**Table 4:** Evidence-based washout recommendations.

Source	Recommended Washout Period	ICI Class/Regimen	Evidence Level
AASLD 2023 [12]	≥90 days (3 months)	All ICIs	Guideline recommendation
Kuo et al. 2023 [77]	≥42 days (1.5 half-lives)	PD-1/PD-L1 (nivolumab, pembrolizumab, atezolizumab; t½ ~28 days)	Retrospective analysis
Guo et al. 2024 [59]	≥30 days	Heterogeneous PD-1 inhibitors ± TKIs ± LRT	Multicenter cohort (OR = 0.096)
Ma et al. 2025 [57]	>30 days or >1.5 half-lives	All ICIs	Global registry (OR = 0.24–0.36)
ASCO 2025 Meta-analysis [135]	>60 days	All ICI classes (PD-1: 40.6% AR; PD-L1: 0% AR; CTLA-4: 25% AR)	Systematic review (OR = 0.10)
Schnickel et al. 2022 [137]	>90 days	PD-1 (nivolumab monotherapy)	Single-center series
Wassmer et al. 2023 [138]	≥90 days	All ICI classes (PD-1/PD-L1/CTLA-4), pre- and post-LT settings	Narrative review

Abbreviations: ICI, immune checkpoint inhibitor; PD-1, programmed cell death protein 1; PD-L1, programmed death-ligand 1; CTLA-4, cytotoxic T-lymphocyte antigen-4; TKI, tyrosine kinase inhibitor; LRT, locoregional therapy; LT, liver transplantation; AR, acute rejection; OR, odds ratio; t½, half-life.

## Data Availability

No new data were created or analyzed in this study.

## Abbreviations

The following abbreviations are used in this manuscript:

HCC	Hepatocellular carcinoma
LT	Liver transplantation
ICIs	Immune checkpoint inhibitors
TACE	Transarterial chemoembolization
TARE	Transarterial radioembolization
SBRT	Stereotactic body radiotherapy
BCLC	Barcelona Clinic Liver Cancer
mRECIST	Modified Response Evaluation Criteria in Solid Tumors
AFP	Alpha-fetoprotein
MELD	Model for End-Stage Liver Disease
MASLD	Metabolic dysfunction-associated steatotic liver disease
HBV	Hepatitis B virus
HCV	Hepatitis C virus
ORR	Objective response rate
PFS	Progression-free survival
OS	Overall survival
RFS	Recurrence-free survival
TME	Tumor microenvironment
irAEs	Immune-related adverse events
PD-1	Programmed death-1
PD-L1	Programmed death-ligand 1
CTLA-4	Cytotoxic T-lymphocyte-associated protein 4
UCSF	University of California, San Francisco
RETREAT	Risk estimation of tumor recurrence after transplant
